# Longitudinal Changes on Optical Coherence Tomography Angiography in Retinal Vein Occlusion †

**DOI:** 10.3390/jcm10071423

**Published:** 2021-04-01

**Authors:** Swetapadma Tripathy, Hong-Gam Le, Maria Vittoria Cicinelli, Manjot K. Gill

**Affiliations:** 1 Department of Ophthalmology, Feinberg School of Medicine, Northwestern University, 645 N Michigan Avenue, Ste 440, Chicago, IL 60611, USA; SwetaTripathy@gmail.com (S.T.); hgle888@gmail.com (H.-G.L.); cicinelli.mariavittoria@hsr.it (M.V.C.); 2 School of Medicine, Vita-Salute San Raffaele University, 20132 Milan, Italy; 3 Department of Ophthalmology, IRCCS San Raffaele Scientific Institute, 20132 Milan, Italy

**Keywords:** retinal vein occlusion, optical coherence tomography angiography, anti-vascular endothelial growth factors, vessel density

## Abstract

Background: To evaluate the longitudinal changes on optical coherence tomography angiography (OCTA) in retinal vein occlusion (RVO). Methods: Retrospective study of patients with RVO treated with intravitreal anti-vascular endothelial growth factors (VEGF) for macular edema. Foveal avascular zone (FAZ) area, vessel density (VD), vessel length density (VLD), and adjusted flow index (AFI) were calculated. The unaffected eye of each participant was used as a control. Results: Twelve RVO eyes were included, receiving 6 ± 3 anti-VEGF injections over a follow-up of 10.4 ± 3.1 months. Compared to fellow eyes, RVO eyes had lower VD and VLD at inclusion (*p* = 0.07 and *p* = 0.04) and last visit (*p* = 0.002 and *p* < 0.001). VD, AFI, and VLD did not change over time, while FAZ area increased in RVO eyes (+0.016 ± 0.024 mm^2^, *p* = 0.04). AFI correlated with duration of disease (*r* = 0.63, *p* = 0.02). Visual acuity was inversely related to VD and VLD over the follow-up. Conclusions: OCTA parameters remained stable with sustained anti-VEGF treatment in RVO, while changes in the FAZ area may suggest capillary remodeling after RVO.

## 1. Background

Retinal vein occlusion (RVO) affects 16 million people worldwide and is the second most common retinal vascular disease [1]. Vision loss may occur due to macular edema (ME) or neovascular complications, which can only be partially predicted by the extent of retinal capillary nonperfusion [2,3]. The use of anti-vascular endothelial growth factor (VEGF) agents currently represents the first-line of treatment for ME secondary to RVO [4,5,6,7]. 

Conventionally, fluorescein angiography (FA) is used to assess the degree of peripheral and macular ischemia; nevertheless, FA is time-consuming and has the potential for allergic side-effects [8]. Optical coherence tomography angiography (OCTA) offers a rapid and noninvasive alternative to image capillary nonperfusion; OCTA also provides high resolution, three-dimensional visualization of the different layers of the retinal vasculature [9,10]. Limitations of OCTA in the study of RVO eyes include the risk of masking artifacts by vitreal and intraretinal hemorrhages, intraretinal fluid, and exudates; the risk of moving artifacts in eyes with poor fixation; and the small field of view of currently available commercial OCTA systems, which confine imaging within the posterior pole.

Many studies have examined OCTA parameters in patients suffering from RVO, most being limited by a cross-sectional design [11,12,13,14,15,16]. Only a few papers have been published regarding the foveal avascular zone (FAZ) size and vessel density (VD) changes after treatment with anti-VEGF, showing contradictory results [17,18,19,20]. Because there is an association between VEGF upregulation and ischemia, it could be hypothesized that anti-VEGF treatments may affect capillary perfusion both in the posterior pole and the retinal periphery. Retinal reperfusion after anti-VEGF treatment is controversial [21]. Previous studies reported a decrease in areas of retinal nonperfusion in patients with RVO after VEGF inhibition [22,23]. Longitudinal studies with OCTA may help in disentangling this issue.

The purpose of this study was to examine the longitudinal OCTA changes in RVO patients, focusing on vessel length density (VLD) [24,25], which eliminates the disproportionate representation of large-caliber vessels, and adjusted flow index (AFI), a relative measure of flow velocity [26,27,28]. The secondary outcome was to correlate these parameters with the demographic and clinical characteristics of the study population, including the number of anti-VEGF agents administered for ME. 

## 2. Methods

### 2.1. Study Population 

Electronic medical records of patients with a history of central (CRVO) or branch RVO (BRVO) with ME, referred to the Department of Ophthalmology of Northwestern University between January 2016 and March 2018, were reviewed. The diagnosis of CRVO or BRVO was confirmed by a retinal specialist (M.G.) using multimodal imaging. All the eyes underwent a variable course of anti-VEGF intravitreal injections before inclusion in the study; none had received intravitreal steroids before inclusion. None of the eyes were clinically refractory to intravitreal injections of anti-VEGF.

Patients with at least 6 months of follow-up were included. The first OCTA performed after the resolution of ME following anti-VEGF therapy was considered as the baseline. The last OCTA available without ME was taken as the end of the follow-up to reduce the artifacts on OCTA quantification. 

Exclusion criteria were: (1) ME secondary to causes other than RVO (e.g., diabetic macular edema, age-related macular degeneration, postsurgical macular edema); (2) significant media opacity; (3) history of ocular trauma or surgery ≤ 6 months before inclusion; (4) uncontrolled glaucoma, i.e., progressive visual field loss and/or intraocular pressure (IOP) > 25 mmHg despite maximal antiglaucoma treatment in the study eye; (5) bilateral RVO. The presence of any ocular disease or media opacity in the fellow eye was another criterion for exclusion.

### 2.2. Study Procedures

At the time of inclusion, all the individuals underwent best-corrected Snellen visual acuity (BCVA), IOP measurement, slit-lamp examination, and indirect ophthalmoscopy as per the standard of care. Each patient also underwent SD-OCT on Spectralis HRA (Heidelberg Engineering; Heidelberg, Germany) and OCTA (RTVue-XR Avanti, Optovue, Inc., Fremont, CA, USA) in both the RVO and the fellow eye [29]. Demographic (age, gender) and clinical data (duration of RVO, previous treatments) were extracted from the patients’ charts.

Patients were followed based on physician recommendations. The SD-OCT and the OCTA were repeated at each visit. In the case of ME recurrence, RVO eyes were treated with a variable number of intravitreal injections of bevacizumab, aflibercept, or ranibizumab administered at a minimum of 4-week intervals between baseline and conclusion of the study with a pro-re-nata approach. No corticosteroid injections were used after inclusion. The number of injections performed before and after inclusion in the study was collected for each eye.

### 2.3. OCTA Measurements

Manually segmented angiographic full-retinal-thickness slabs from 3 μm beneath the ILM to the middle of the outer nuclear layer were exported and analyzed using Image J (Fiji) [30]. Layers’ segmentation at the internal limiting membrane and the outer plexiform layer was confirmed by two trained ophthalmologists (H-G.L. and S.T.). The full-thickness OCTA scans were analyzed to avoid any potential bias in layers’ segmentation due to residual subclinical intraretinal thickening, intraretinal exudates, or disorganization of retinal inner layers which may persist after anti-VEGF treatment [31]. Only OCTA images with no significant artifacts and a signal strength index above 50 were considered for the analysis. 

**Figure 1:**
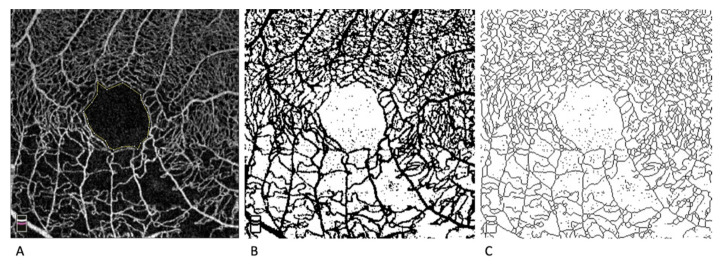
Optical coherence tomography (OCTA) image processing. (**A**) 3 × 3 mm OCTA image centered on the fovea in an eye with central retinal vein occlusion (CRVO) and previously treated macular edema. The foveal avascular zone area (FAZ) was manually traced (*yellow line*). (**B**) Binary conversion of the same image used to measure the vessel density (VD). (**C**) Binarized and skeletonized image used to measure the vessel length density.

The same two individuals manually traced and calculated the FAZ area in mm^2^. The VD was calculated as the percentage of the area occupied by vessel pixels over the total area after image binarization [26]. The VLD was calculated similarly, but skeletonization of the slab was performed after binarization (Figure 1) [32]. The VLD was computed as the ratio of vessel length (mm) over the total area in mm^2^. The AFI was obtained through a global threshold technique, as previously described [24].

Left: 3 × 3 mm OCTA image centered on the fovea in an eye with central retinal vein occlusion and previously treated macular edema. The foveal avascular zone area was manually traced. 

Center: Binary conversion of the same image to measure the vessel density. 

Right: Binarized and skeletonized image to measure the vessel length density. 

### 2.4. Statistical Analysis 

Statistical calculations were carried out with the open-source programming language R. The cutoff point for statistical significance was *p* < 0.05. Descriptive statistics were reported as the mean ± standard deviation (SD) or median and interquartile range (IQR) for continuous variables and frequency and proportion for categorical variables. The BCVA was expressed in LogMAR for statistical calculations. The fellow eyes were used as paired controls.

A two-way intraclass correlation coefficient (ICC) was to measure the strength of inter-rater agreement between the two readers in measuring the FAZ area.

OCTA parameters (VD, VLD, FAZ area, and AFI) between the inclusion and the last follow-up visit were compared with a paired *t*-test, as the difference of pairs followed a normal distribution for each tested variable. At both time points, the same variables were compared between study and control eyes; a linear mixed model was designed, using the condition (RVO or control) as a fixed factor and the patient identification number as a random factor. The BCVA across follow-up was investigated similarly. 

A Pearson correlation coefficient was used to evaluate the relationship between the number of injections with the duration of RVO and between the OCTA parameters and the BCVA. The same analysis was used to investigate the effect of demographic and clinical features on VD, VLD, FAZ area, and AFI.

Finally, the absolute increase in the FAZ area in RVO eyes was measured as the difference between the FAZ area at the final visit and the one at the first available OCTA. The factors potentially affecting FAZ enlargement were investigated with univariable linear models.

## 3. Results

Ten eyes with CRVO and two eyes with BRVO were included. The demographic and clinical characteristics at the time of inclusion are summarized in Table 1. The study cohort varied widely in terms of duration of disease (range: 1–1341 months) and the number of anti-VEGF administered before enrollment (range: 1–70). The duration of RVO did not correlate with the number of injections previously received (*p* = 0.2). 

The median follow-up between the inclusion and the last visit was 10 months. The RVO eyes underwent a median of 5 (range: 2–12) additional anti-VEGF injections during the follow-up period; again, no correlation was found between the duration of RVO and the number of injections received during the study period (*p* = 0.4).

The visual acuity at both inclusion (*p* = 0.01) and the last follow-up visit (*p* = 0.004) was significantly worse in RVO eyes compared to controls (Table 2). There were no statistically significant changes in the BCVA between the inclusion and the last follow-up visit (*p* = 0.4 and *p* = 0.3 for RVO and control eyes, respectively).

### 3.1. OCTA Parameters

The two readers showed excellent agreement (ICC = 0.9) in the FAZ area measurements; therefore, the readings from Reader 1 were considered in the analysis. Compared to the fellow eyes, RVO eyes had lower VD at the last visit (*p* = 0.002) and lower VLD at both the inclusion (*p* = 0.04) and the last visit (*p* < 0.001) (Table 2); the VD (*p* = 0.8 and *p* = 0.2) and the VLD (*p* = 0.8 and *p* = 0.06) remained unchanged over time in both study and control eyes.

There was no difference in the FAZ area between RVO and control eyes at baseline (*p* = 0.1). The FAZ area increased slightly in the RVO eyes (+0.016 ± 0.024 mm^2^, *p* = 0.04), while it remained stable in the fellow eyes (*p* = 0.5). At the last visit, there was a 0.10 mm^2^ difference between the two groups, albeit nonsignificant. None of the demographic and clinical factors investigated, including age, gender, number of anti-VEGF injections administered before inclusion, number of anti-VEGF administered after inclusion, size of the FAZ, VD, VLD, and AFI at inclusion, and length of follow-up, had a significant impact on FAZ enlargement on univariable linear models.

The AFI was comparable between RVO and control eyes at inclusion (*p* = 0.6) and last follow-up (*p* = 0.8) and did not show any longitudinal change (*p* = 0.6 in both RVO and control eyes).

There was a tendency towards larger FAZ and worse BCVA in patients with a longer history of RVO at study enrollment, despite not reaching statistical significance (*p* = 0.07 in both cases, Table 3). A positive association was found between the AFI and the duration of disease (*r* = 0.63, *p* = 0.02). Conversely, the VD and VLD parameters did not correlate with the number of anti-VEGF injections received at the inclusion or last-follow-up visit (Table 3).

### 3.2. Visual Acuity and OCTA Parameters

**Figure 2:**
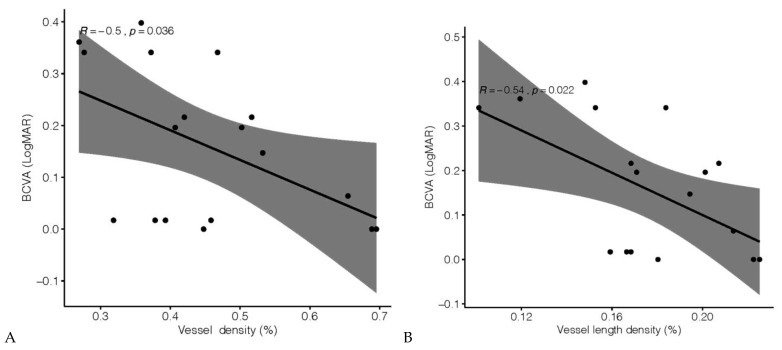
Linear correlation between best-corrected visual acuity (BCVA) and optical coherence tomography (OCTA) parameters at the inclusion date. (**A**) Linear correlation between BCVA and vessel density (VD). (**B**) Correlation between BCVA and vessel length density (VLD). See Methods (Section 2) for more details on these parameters.

The BCVA at the inclusion visit correlated with VD (*r* = −0.5, *p* = 0.04) (Figure 2A) and VLD (*r* = −0.54, *p* = 0.02) (Figure 2B) but not with the FAZ area (*r* = 0.2, *p* = 0.4) or the AFI (*r* = 0.14, *p* = 0.6). The visual acuity at the end of the follow-up still had a linear relationship with VD (*r* = −0.7, *p* < 0.001) and VLD (*r* = −0.8, *p* < 0.001) but also with the FAZ area (*r* = 0.5, *p* = 0.04). The BCVA was unrelated to the time elapsed from the RVO onset and the number of anti-VEGF injections administered (Table 2).

Panel A shows the vessel density, while Panel B shows the vessel length density (see Methods (Section 2) for detailed information about these parameters)

## 4. Discussion

In this study, we longitudinally analyzed 12 eyes with RVO who received anti-VEGF for ME before inclusion and during the follow-up. To date, this is one of the first papers reporting longitudinal data on VLD and AFI in eyes with RVO. We found a progressive enlargement of the FAZ area over time, while the other OCTA parameters (VD, VLD, and AFI) remained stable. None of the variables studied influenced the entity of FAZ enlargement. The visual acuity in RVO eyes did not vary significantly once the ME had resolved, and it was linearly related to the capillary density at different timepoints. 

A positive association was found between the AFI and the duration of disease at the end of the study period. AFI is an approximate measure of blood flow velocity based on pixel intensity, which has been shown to correlate with flow velocity in OCTA within a limited range [28]. Although the AFI values did not change within the study period, we hypothesize that blood flow velocity tends to normalize with time.

In our cohort of patients, we noticed an increase in the FAZ area over 10 months, despite no significant changes in the perifoveal vascular perfusion. The FAZ remained unchanged in the nonaffected fellow eyes. Our results are in parallel with those from Suzuki et al., who reported an increase in the FAZ area in eyes with RVO over six months after initiation of anti-VEGF therapy [17]. FAZ remodeling may suggest dynamic changes in the vascular flow in eyes with RVO; we hypothesize these changes become more evident in the FAZ before affecting the whole capillary density of the OCTA slab. Nevertheless, the retrospective, uncontrolled nature of our study does not allow for the investigation of the underlying causes of FAZ enlargement.

Our data showed no difference in the FAZ area between RVO and fellow eyes at the inclusion visit. This is apparently in contrast with the previous literature, as most of the cross-sectional studies reported an enlarged FAZ in RVO eyes [33]. One reason for this discrepancy is that in eyes with RVO, the measure of the FAZ could be affected by the presence of ME, which makes it falsely larger as the intraretinal cysts displace the central capillary network. The patients included in our study did not demonstrate ME at the time of the first OCTA exam, to avoid segmentation artifacts by intraretinal exudation [34]. Another possible explanation is a selection bias in favor of patients with better visual function and better fixation during OCTA acquisition at baseline as the FAZ size is inversely related to the visual acuity [11,35]. Finally, we cannot exclude that the lack of a significant difference at baseline could be related to regression towards the mean phenomenon [36].

While all previous studies agree on significant capillary dropout occurring in eyes with RVO compared to healthy controls [12,15,16,33,37,38], the data from longitudinal reports are controversial. In particular, the effect of anti-VEGF therapy on retinal perfusion is unclear, as some reports suggest it may induce vasoconstriction [39,40], while others show that it may improve the state of retinal perfusion [41]. A subanalysis of fluorescein angiograms in patients participating in the BRAVO and CRUISE studies showed that patients in the sham group had progression of retinal nonperfusion, in contrast to those in the anti-VEGF treatment groups. Thus, the authors concluded that monthly injections of ranibizumab could prevent the worsening of retinal nonperfusion [22]. 

By using OCTA, Suzuki et al. reported an improvement in the nonperfused areas after anti-VEGF therapy, with more frequent injections resulting in a greater reduction in ischemic areas [17]. Similarly, Ciloglu et al. found a significant increase in parafoveal VD values after anti-VEGF treatment [42]. We found no changes in VD and VLD with anti-VEGF treatment over the follow-up period, in accordance with previous reports [18,43]. As data on OCTA capillary density in eyes with RVO in the absence of treatment are not made available, it is difficult to identify the real impact of anti-VEGF on longitudinal OCTA parameters. We can hypothesize that anti-VEGF therapy may prevent the progression of capillary nonperfusion; however, the absence of a control arm does not allow us to confirm this hypothesis. The inclusion of patients with subacute RVO may give support to our findings. In fact, the presence of intraretinal hemorrhages and venous congestion at the onset of RVO may artifactually obscure the retinal capillaries, thereby reducing the VD. As a consequence, VD values are found falsely increased once RVO signs resolve. 

We observed a slight change in the VD of fellow eyes on longitudinal follow-up. An intervisit coefficient of variation for measurements of VD up to 4.9% and 6.8% has been reported in healthy and diseased eyes, respectively. This suggests that, although OCTA has good reproducibility, a minimal variation between repeated scans should be expected [44].

We demonstrated poor perfusion (VD and VLD) was inversely related to the BCVA, in agreement with previous studies [12]. Winegarner et al. found that a better VD post-treatment was significantly associated with a higher BCVA score at 12 months [18]. Persistent retinal capillary ischemia may cause irreversible damage to the retinal tissue and function, resulting in irreversible visual impairment. Patients with higher levels of retinal nonperfusion may be counseled appropriately regarding the limited prognosis of visual recovery, even after the resolution of ME. Despite continuous anti-VEGF treatment, we did not observe significant changes in the visual acuity in RVO eyes through the course of the follow-up period. The majority of the study eyes had resolved ME at both the first and the last recorded visit and had already received anti-VEGF before inclusion. A “ceiling” effect with respect to further improvement in the visual function should be considered. The relative stability in visual acuity after continuous anti-VEGF injections is consistent with the long-term follow-up of randomized clinical trials of anti-VEGF in RVO, which showed maintenance of the visual function following the first year of treatment [45].

Finally, the patients in our cohort underwent a significantly higher number of anti-VEGF injections compared to the study from Winegarner et al. (3.7 ± 1.4 injections in 12 months) [18] and Tsuboi and Kamei (2.6 ± 1.8 in 12 months) [43]. Persistence of ME in eyes with RVO has been associated with the extent and the progression of posterior pole nonperfusion both on fluorescein angiography [21] and OCTA [46]. As a consequence, a greater number of intravitreal injections have been reported in eyes with a higher level of capillary disruption [46]. Interestingly, we found no correlation between the number of anti-VEGF injections and the OCTA parameters, including VD and VLD. The difference in the study design (i.e., the inclusion of more long-standing RVO) and a relatively short follow-up may account for this discrepancy.

The strengths of our study include the longitudinal design and the exclusion of ME on OCTA, which may increase the reliability of segmentation and our measurements. On the other hand, our study has limitations. We did not analyze factors associated with recurrence of ME during follow-up [20] and are unable to predict whether eyes with worse macular perfusion parameters are more prone to ME. Moreover, we did not analyze the peripapillary area, which may have led to different results, as a prospective study including 18 CRVO patients found increased peripapillary VD after anti-VEGF therapy [47]. We also did not consider healthy eyes as controls but we used the fellow eyes. As patients share the same systemic risk factors (hypertension, diabetes, or coagulation disorders), the retinal perfusion of the contralateral eye may be affected even in absence of visible retinal pathology. For instance, Adhi et al. showed that the FAZ was larger, and the VD was lower in the fellow eyes of patients with RVO compared to healthy subjects [33]. Nevertheless, we were interested in the longitudinal changes of OCTA parameters rather than their cross-sectional evaluation. Finally, the small sample size and the large differences in the duration of RVO, the number of injections, and the length of the follow-up represent potential confounders to our study. 

## 5. Conclusions

We found no significant longitudinal change in the OCTA parameters of AFI, VD, and VLD in RVO eyes undergoing anti-VEGF therapy. Conversely, the FAZ area tended to enlarge over time, with a larger area associated with worse visual acuity. The VD and the VLD were prognostic predictors of the visual outcome after the resolution of ME, and this correlation was maintained over the follow-up period. We speculate that anti-VEGF therapy might play a role in preserving the global macular perfusion in RVO eyes. Future studies with a larger sample size and a longer follow-up duration exploring a larger area of the posterior pole with wide-field OCTA are warranted.

## Figures and Tables

**Table 1:** Demographic and clinical characteristics at inclusion in the study.

Diagnosis (%)	
CRVO	10 (83)
BRVO	2 (17)
Gender (%)	
Male	5 (58)
Female	7 (42)
Age (median, IQR, years)	72, 62–80
Hypertension (%)	9 (75)
Diabetes (%)	3 (25)
Duration of RVO (median, IQR, months)	17, 10–68
Duration of follow-up (median, IQR, months)	10, 9–13
Anti-VEGF agent injections before inclusion (median, IQR)	9, 6–16
Anti-VEGF agent injections during follow-up (median, IQR)	5, 3–8

BRVO: branch retinal vein occlusion; CRVO: central retinal vein occlusion; IQR: interquartile range; VEGF: vascular endothelial growth factor.

**Table 2:** Changes in optical coherence tomography angiography parameters and best-corrected visual acuity (BCVA) in retinal vein occlusion (RVO) and control eyes.

	RVO Eyes		Control Eyes		RVO vs. Control Eyes	
	Inclusion Visit	Last Follow-Up	Inclusion Visit	Last Follow-Up	*p*-Value (Inclusion Visit)	*p*-Value (Last Follow-Up)
BCVA (LogMAR)	0.23 ± 0.13	0.26 ± 0.13	0.02 ± 0.02	0.04 ± 0.03	0.01 *	0.004 *
	0.4		0.3			
FAZ area (mm^2^)	0.41 ± 0.18	0.43 ± 0.19	0.34 ± 0.15	0.33 ± 0.16	0.1	0.07
	0.04 *		0.5			
VD (%)	41.4 ± 8.65	41.9 ± 7.49	57.1 ± 16.9	66.5 ± 16.7	0.07	0.002 *
	0.8		0.2			
VLD (mm^−1^)	16.5 ± 3.16	16.3 ± 2.91	19.5 ± 2.9	21.8 ± 1.62	0.04 *	<0.001 *
	0.8		0.06			
AFI (%)	44.9 ± 4.48	44.2 ± 5.25	44.4 ± 3.2	43.6 ± 4.51	0.6	0.8
	0.6		0.6			

FAZ: foveal avascular zone, VD: vessel density, VLD: vessel length density, AFI: adjusted flow index. * statistically significant *p*-value.

**Table 3:** Correlation of OCTA parameters and best-corrected visual acuity (BCVA) with the duration of retinal vein occlusion (RVO) and the number of anti-vascular endothelial growth factor (VEGF) received.

	Inclusion Visit		Last Follow-Up	
OCTA Parameter	Time from RVO	Anti-VEGF Injections Received ^†^	Time from RVO	Anti-VEGF Injections Received ^††^
FAZ area (mm^2^)	*R* = 0.54	*R* = −0.07	*R* = 0.50	*R* = −0.12
	*p* = 0.07	*p* = 0.8	*p* = 0.1	*p* = 0.7
VD (%)	*R* = −0.07	*R* = −0.20	*R* = −0.16	*R* = 0.06
	*p* = 0.8	*p* = 0.5	*p* = 0.6	*p* = 0.8
VLD (mm^−1^)	*R* = −0.03	*R* = −0.12	*R* = −0.02	*R* = 0.003
	*p* = 0.9	*p* = 0.7	*p* = 0.9	*p* = 0.9
AFI (%)	*R* = 0.49	*R* = 0.09	*R* = 0.63	*R* = 0.06
	*p* = 0.1	*p* = 0.8	*p* = 0.02 *	*p* = 0.9
BCVA (LogMAR)	*R* = −0.53	*R* = −0.24	*R* = −0.16	*R* = −0.13
	*p* = 0.07	*p* = 0.4	*p* = 0.6	*p* = 0.6

^†^ Number of anti-VEGF received before the inclusion date. ^††^ Number of anti-VEGF received at the last follow-up visit. * Statistically significant *p*-value.

## Data Availability

The datasets used and/or analyzed during the current study are available from the corresponding author on reasonable request.
